# Relationship between occlusal parameter (OP) using T-scan and temporomandibular disorder (TMD)

**DOI:** 10.6026/973206300223180

**Published:** 2026-05-31

**Authors:** Tejaswini Patil, Avinash Kshar, Raghavendra Byakodi, Arati Paranjpe, Sunil Awale, Manishkumar Shete

**Affiliations:** 1 Department of Oral Medicine & Radiology, Vasantdada Patil Dental Collage & Hospital, Sangli, Maharasthra, India

**Keywords:** Malocclusion, T-SCAN, temporomandibular disorder (TMD), protrusion, right canine, clinical diagnosis

## Abstract

In case of malocclusion, along with clinical diagnosis, radiographic techniques are needed due to the complex anatomy and pathology. Therefore, it is of interest to evaluate the relation between OP using T-SCAN and TMD. We have included 60 patients that were divided in group A & B in equal parts. Here group patients were clinically diagnosed patients and group B as control group, respectively. We found that, only protrusion and right canine had high significant difference between the 2 groups. Thus, we show that the relation can be best indicated using new technologies like T-scan. Furthermore large prospective studies over long period are required for more definitive and confirmatory result.

## Background:

Occlusion optimization is essential, as it facilitates proper oral functions, enhances aesthetic appearance and contributes to disease prevention [1]. Malocclusion refers to any deviation from normal occlusion in the mouth [1]. It can have an effect on dental diseases, mastication, phonation, aesthetics (particularly facial profile) and other oral cavity functions [2]. In addition to above, it also includes a comprehensive system for managing patient files that facilitates the storage of patient records and the tracking of occlusal scans, rendering it an essential element of the clinical workstation for supporting the diagnosis and treatment [3]. Therefore, it is of interest to report and evaluate the relationship between occlusal parameter (OP) using T-scan & TMD.

## Materials and Methods:

A cross-sectional observational convenience type of sampling study was conducted in the Department of Oral Medicine & Radiology with a sample size of 60 patients between 15 to 50 years. They were further divided into group A & B in equal parts. Where group A patients were clinically diagnosed for signs & symptoms of TMD and fulfilling DC/TMD criteria and group B (control group), respectively.

## Materials:

[1] Sterile kidney tray

[2] Autoclaved Mouth Mirror, straight Probe, Explorer, Tweezer & Williams graduated probe.

[3] Sterile Vernier caliper & gauze pieces.

[4] Cheek retractor

[5] Cotton holder & waste receiver.

[6] Non-woven surgeon faces mask & cap.

[7] Examination gloves

[8] Ordinary denatured spirit

[9] Stethoscope

[10] T-scan device, software and sensor ( small & large)

[11] Monitor

[12] Sensor holder (small & large) 

## Methods:

## Comprehensive patient evaluation:

Furthermore, mandible movements were observed to determine whether or not there were any constraints or abnormalities. A more thorough examination may be performed by placing a stethoscope over the joint region over the lateral mouth surfaces while opening and shutting the mouth to record clicking or crepitation sounds. The anterior region was palpated above the zygomatic arch and anterior to the TMJ, middle region, above the TMJ and superior to arch and finally, posterior region, above and behind the ear. Moreover, patient was asked to clench the teeth, followed by whether it hurts or is just uncomfortable, further finger was placed on zygomatic to palpate masseter and the response was recorded according to the visual analog scale (VAS). Additionally, the intraoral finger was moved up the anterior border of the ramus, coronoid process and towards tendon to detect if there is any discomfort or pain. In addition to this, painful inferior lateral pterygoid muscle was checked by pushing mandible forward against resistance while medical pterygoid muscle was checked by opening upto 10-15mm from intercuspal contact and was checked by VAS.

## Occlusal parameter evaluation method:

Evaluation and visualization of occlusal contacts, forces, time and their parameters were carried out in both the treatment and control groups using a T-scan, a holder and a U-shaped pressure-sensitive gauge in conjunction with a computer.

## Recording technique:

Patient was asked to seat in upright, forward looking position with mandible parallel to floor. Recording handle with sensor & sensor support is placed into patient mouth with sensor support pointer between the maxillary CI, keeping the scanning handle parallel to occlusal plane and it is initiated by pressing button which lets to formation of arch model automatically on screen. Further patient is asked to close the mount until complete interception without any excursive movements.

## Data interpretation:

The recorded data is shown as a force film, whereby the center of force trajectory illustrates the historical course of the force's center from the beginning of the recording to the currently displayed frame. The relative occlusal force is shown in three-dimensional colored bar graphs. The hue and elevation of each bar represent the magnitude of force per contact. The intensity of the occlusal load is shown by color coding, with red indicating the greatest force and blue denoting the smallest force. Consequently, by acquiring knowledge on the first occlusal contact, it may be modified, enabling the establishment of simultaneous occlusal contact. The outcome of this occlusal treatment is that the patient has a more extensive contact sensation, as the T-scan facilitates the formation of accurate and quantifiable bilateral simultaneous occlusal contacts. Left and right sides to be shown in different colour codes (green on the left and red on the right).

## A-D vertical line of T-scan:

[1] A: Initial occlusal contact.

[2] B: Where all the teeth have intercepted during closure.

[3] A-B: Time taken from initial tooth contact to maximum intercuspation. This is represented as occlusion time (OT) and should ideally be under 0.2 s; longer OT indicates more interference and premature contacts during closure.

[4] B-C: Time taken when the teeth are intercepted during closure.

[5] C: Commencement of discussions D: All the posterior teeth have disclosed and guidance is achieved.

[6] C-D: Represents disillusion time (DT). During excursion, the DT should be under 0.4 s; longer DT indicates more occlusal surface frictional contacts during excursion.

## Horizontal line (T-scan):

[1] Black line: Describes the total force changes throughout entire scan.

[2] Red line: Describes the force changes on right side of the arch, throughout entire scan.

[3] Green line: Describes the force changes on left side of the arch, throughout entire 

## Interpretation of Occlusion and disclusion time:

According to the manufacturer of T-scan, OT is recommended as less than 0.2s and DT less than 0.4 s. Prolonged OT and DT are referred to as functional disorders of the masticatory system.

## Inclusion criteria:

[1] All clinically diagnosed cases of TMD ( according to DC/TMD criteria, 2014)

[2] Male and female

[3] Age between 15 to 50 years 

[4] In control group, people with good oral hygiene, no TMD involvement and those who not received TMD treatment. 

## Exclusion criteria:

[1] Patients with history of trauma or surgery

[2] Those who currently use medication that might mask TMD symptoms

[3] People with trigeminal neuralgia, burning mouth syndrome, migraine, atypical facial pain and patients with reduced mouth opening due to OSMF, scleroderma, rheumatoid arthritis and congenital anomalies of TMJ.

## Statistical analysis:

For assessing qualitative data tools of descriptive statistic was used while chi-square test was used to find differences among the groups respectively. Furthermore, unpaired - t- test was used to determine mean of two groups of unrelated subjects.

## Results:

In Table 1, we have found that, in both the groups majority of the patients were females with 18 in number (60%) than males respectively. Thus showed non-significant difference between the 2 groups. Moreover, Table 2, Table 3 also showed non- significant difference between the 2 groups. Table 4 showed no deviation for all 30 patients who were in group B while 8 patients showed deviation to right side and 9 showed deviations to left side in group A. Thus, there was a statistically high significant difference between the 2 groups respectively. Table 5 showed no jaw deflection patient in both the groups. Table 6 showed only 7 patients showed clicking sound on right side, 6 patients on left side and only 5 patients of both sides respectively in group A while no patients showed any signs and symptoms in group B . Thus, there was a statistically high significant difference between the 2 groups. Table 7 showed no creptius patient in both the groups. Table 8 showed that, the pain score was found the majority of the patients had medial pterygoid muscle with 1.333 ± 1.21, followed by masseter muscle with 0.966 ± 0.99, temporalis muscle with 0.933 ± 1.08, and finally lateral pterygoid muscle with 0.866 ± 1.0, respectively, in group A. While on the other hand, group B did showed any sign and symptoms for pain to record. Thus, there was statistically high significant difference found between the 2 groups for all the muscles. Table 9 showed that, there was statistically non-significant difference between the 2 groups for occlusal forces on left, right and AOF as the p value was 0.384, 0.317 and 0.809 respectively. Table 10 showed that, only protrusion and right canine had high significant difference between the 2 groups while occlusion time, disclusion time and left canine guidance showed non-significant difference.

## Discussion:

Recent research underscores the biopsychosocial aspects of prevalent painful TMD, including myalgia and/or arthralgia and highlights their interrelations with overall health. TMD-pain represents the most prevalent form of non-odontogenic orofacial pain and serves as a s ignificant impetus for individuals to seek treatment, contributing to increased healthcare expenditures and diminished quality of life in those affected by it [4]. In our study we found that, majority of the patients were females than males. A study found that, TMD are a set of illnesses that cause pain in the TMJ (arthralgia, arthritis) and/or pain in the masticatory muscles (myofascial TMD). Particularly masticatory muscle pain, the author suggested that psychological stresses are also thought to contribute to the development of pain associated with TMJ disorders. When it comes to total joint dysfunction (TMD), women are far more likely to suffer from it than men. One possibility for the greater frequency of this illness in women has been suggested to be the female sex hormone estrogens. This is despite the fact that there are potentially several explanations for variations in the prevalence of TMD that are tied to gender [5]. Furthermore, we have found, 8 patients showed deviation to right side and 9 showed deviation to left side in group awhile in another study, the researchers found that right side deviation was seen in 11 patients, left side in 14 and non-deviation in total of 10 patients. Thus, concluded that, the TMJ on the deviated side showed a significantly steeper eminence than that on the non-deviated side [6]. In our study, 7 patients showed clicking sound on right side, 6 patients on left side and only 5 patients of both sides respectively in group A. In another study researchers concluded that among the 41% of patients who had TMJ disorders, the most common TMJ pathology was clicking which was 89% and most patients had either bilateral clicking (32%) or clicking on the right side (32%) and left side was 24% respectively [7]. In our study, the majority of the patients had medial pterygoid muscle with 1.333 ± 1.21, followed by masseter muscle with 0.966 ± 0.99, temporalis muscle with 0.933 ± 1.08 and finally lateral pterygoid muscle with 0.866 ± 1.0, respectively, in group A. A research similar to our results determined that patients in urban clinics had a greater prevalence than those at rural clinics. Palpation of the TMJ revealed moderate to severe pain in 45% of the case patients. 82.5% of cases showed tenderness upon palpation of at least one masticatory muscle. Tenderness to palpation across the muscles of mastication was moderate to severe, occurring in 35% to 45% of cases [8]. In our study, we have found, statistically non-significant difference between the 2 groups for occlusal forces on left, right and AOF as the p value was 0.384, 0.317 and 0.809 respectively and only protrusion and right canine had high significant difference between the 2 groups while occlusion time, disillusion time and left canine guidance showed non-significant difference. Another study done by researchers concluded that, the prevalence of TMD in this university student population was 17%. There were significant associations of TMD with psychological parameters and functional occlusal parameters [9]. Another study in accordance to our study showed that, the type of occlusal indicator influences the registration of contacts and also they observed that T-scan was more reliable and accurate method than the traditional methods to record and achieve a balance occlusion in clinical practice [10]. In a study the researchers found that, the average OT and DT in normal dentate subjects with healthy TMJs were 0.69 s and 0.79 s respectively. DT can be improved on treatment and early detection of the disorder. T scan can be used as an indication and evaluation of progress of the disease [11]. Another similar study to our results showed that mean occlusion time of TMD group was significantly longer than the mean occlusion time of control group. [1]. Furthermore a study had showed that prolonged discussion of OP time (>0.8s) links to TMD pain, therefore selective enameloplasty could reduce it to 0.4s in TMD patients [12]. A study showed that, T-Scan-guided splints lowered occlusal time, discursion time and asymmetry (<10%), boosting mouth opening and VAS pain scores in 36 TMD patient. Another study showed that, there was no statistically significant distinction between the gender-based parameters and right and left joint of Joint Vibration Analysis record in all three groups (P > 0.05). At the beginning of orthodontic treatment, there was no statistical difference in the variables within the group and between the groups. As a result of the comparison between the beginning of treatment (T0) and the 6th month (T1), no significant difference was found between the parameters of the anterior/posterior occlusal force distribution of the right-left quadrant and the force distribution of the working and non-working sides in lateral movements and the occlusion and disclusion time parameters. Maximum intercuspal position left/right (Mxlnt nT SCAN 1) was found to be statistically significant at 6 months in individuals who received fixed orthodontic treatment with extraction compared to the none extraction treatment group (P < 0.05). Thus, they conclude that, T-scan is one of the best devices for the early detection of TMD, especially for people undergoing orthodontic treatment. As it offers fast, non-invasive and repeatable occlusion recording [13]. Moreover, a study showed that, 71 subjects (18%) were diagnosed with pain-related TMD, 52 (13%) with IAD, 6 (1.5%) with DJD and 38 (10%) with headache due to TMD. Gender had a significant correlation with pain-related TMD (p= 0.014, OR= 2.16). Maximum pain free opening had a significant inverse relationship with pain related TMD (p= 0.013, OR=0.94), while midline deviation and corrected deviation mouth opening pattern had a significant correlation with IAD (p= 0.04, 0.02, OR= 1.30, 2.74, respectively). Overbite, midline deviation and pain free opening were significantly associated with unilateral open/close clicking (p= 0.04, 0.05, 0.03, OR= 0.77, 1.31, 0.94, respectively). Thus, there was a minimal clinical significance of the correlation between dental occlusion and TMD [14]. A study showed that, out of 98 articles, 47 articles were found in PubMed, ten from Lilacs and 41 from Web of Science. Clinical trials and concluded that, T-Scan is a non-invasive and relatively inexpensive way to diagnose and treat TMDs. It provides a detailed picture of the jaw’s activity, which can be used to customize treatment for each patient. It is also an effective way to monitor treatment progress and ensure that the patient receives the best possible care. Overall, T-Scan is an essential tool for diagnosing and treating TMDs [15].

## Conclusion:

There were eight types of occlusal interferences, including sliding, balancing, working side, posterior protrusive and RCP-ICP discrepancies, link to TMD and appear as timing/force imbalances on T-scan. Thus, repeated interferences cause muscle incoordination and myospasm, marking TMD's initial stage. Hence, T-scan enables early detection, progression evaluation and demands larger studies with comprehensive TMD parameters for robust results.

## Advancement to knowledge:

This study advances TMD knowledge by demonstrating T-scan's precision in detecting eight specific occlusal interferences (e.g., sliding, balancing, RCP-ICP shifts) as early triggers for muscle incoordination. It also highlights dynamic force/timing imbalances invisible to traditional exams, enabling proactive interventions before myospasm escalates.

## Figures and Tables

**Table 1:** Gender distribution

	**Group A (TMD)**	**Group B (Non -TMD)**		**P value, Significance**
	**n (%)**	**n (%)**	**Chi square test**	
Male	12 (40%)	12 (40%)		P =1.000
Female	18 (60%)	18 (60%)	Chi =0.0	(no significant difference)

**Table 2:** Age distribution

	**Group A (TMD) Mean (SD)**	**Group B (Non -TMD) Mean (SD)**	**Unpaired t test**	**P value, Significance**
Age	25.2 (1.54)	24.96 (1.37)	t = 0.619	P =0.539 (no significant difference)

**Table 3:** Mouth opening comparison

	**Group A (TMD) Mean (SD)**	**Group B (Non -TMD) Mean (SD)**	**Unpaired t test**	**P value, Significance**
Mouth opening	42.13 (1.87)	42.66 (2.52)	t = -0.930	p =0.356 (no significant difference)

**Table 4:** TMJ deviation comparison

	**Group A (TMD) n (%)**	**Group B (Non -TMD) n (%)**	**Chi square test**	**P value, Significance**
No deviation	13 (43.3%)	30 (100%)		P<0.001** (highly significant difference)
Deviation to right side	8 (26.7%)	0 (0%)	Chi = 23.721	
Deviation to left side	9 (30%)	0 (0%)		

**Table 5:** TMJ defection comparison

	**Group A (TMD) n (%)**	**Group B (Non -TMD) n (%)**	**Chi square test**	**P value, Significance**
No deflection	30 (100%)	30 (100%)	Chi = 0.0	P =1.00 (no significant difference)
Deflection to right side	0 (0%)	0 (0%)		
Deflection to left side	0 (0%)	0 (0%)		

**Table 6:** TMJ clicking comparison

	**Group A (TMD) n (%)**	**Group B (Non -TMD) n (%)**	**Chi square test**	**P value, Significance**
No clicking	12/30 (40%)	30 /30 (100%)		
Clicking on right side	7/30 (23.3 %)	0 (0%)	Chi = 17.92	p<0.001** (highly significant difference)
Clicking on left side	6/30 (20 %)	0 (0%)		
Clicking on both sides	5/30 (16.6%)	0 (0 %)		

**Table 7:** TMJ crepitus comparison

	**Group A (TMD) n (%)**	**Group B (Non -TMD)**	**Chi square test**	**P value, Significance**
No crepitus	30 (100%)	30 (100%)	Chi =0.0	P =1.000 (no significant difference)

**Table 8:** Comparison of pain vas score

	**Group A (TMD) Mean (SD)**	**Group B (Non -TMD) Mean (SD)**	**Unpaired t test**	**P value, Significance**
Masseter	0.966 (0.99)	0.0 (0.0)	t = 5.298	p<0.001**
Temporalis	0.933 (1.08)	0.0 (0.0)	t = 4.731	p<0.001**
Medial pterygoid	1.333 (1.21)	0.0 (0.0)	t = 6.021	p<0.001**
Lateral pterygoid	0.866 (1.0)	0.0 (0.0)	t = 4.709	p<0.001**

**Table 9:** Comparison of parameter

	**Group A (TMD) Mean (SD)**	**Group B (Non -TMD) Mean (SD)**	**Unpaired t test**	**P value, Significance**
Occlusal forces on left (%)	50.24 (7.34)	51.94 (7.64)	t = -0.878	p = 0.384
Occlusal forces on right (%)	49.75 (7.34)	47.75 (7.99)	t = 1.009	p =0.317
AOF (%)	55.34 (4.44)	55.65 (5.58)	t = -0.243	p =0.809

**Table 10:** Comparison

	**Group A (TMD)**	**Group B (Non -TMD)**		**P value, Significance**
	**Mean (SD)**	**Mean (SD)**	**Unpaired t test**	
Occlusion time (in seconds)	0.389 (0.26)	0.284 (0.294)	t = 1.458	p =0.150
Disclusion time (in seconds)	0.227 (0.213)	0.252 (0.195)	t = -0.473	p =0.638
Protrusion	1.62 (1.631)	0.443 (0.11)	t = 3.938	p<0.001**
Right canine guidance	1.8 (1.09)	0.5 (0.28)	t = 6.293	p<0.001**
Left canine guidance	1.784 (1.09)	0.622 (0.48)	t =5.330	p =0.248
